# Lid mobility in lipase SMG1 validated using a thiol/disulfide redox potential probe

**DOI:** 10.1002/2211-5463.12059

**Published:** 2016-04-13

**Authors:** Shaohua Guo, Grzegorz Maria Popowicz, Daoming Li, Dongjuan Yuan, Yonghua Wang

**Affiliations:** ^1^ School of Light Industry and EngineeringSouth China University of TechnologyGuangzhouChina; ^2^ Institute of Structural BiologyHelmholtz Zentrum MünchenDeutsches Forschungszentrum für Gesundheit und Umwelt (GmbH)NeuherbergGermany

**Keywords:** activation mechanism, disulfide bond, lid, redox potential, redox‐switch, SMG1

## Abstract

Most lipases possess a lid domain above the catalytic site that is responsible for their activation. Lipase SMG1 from *Malassezia globose* CBS 7966 (*Malassezia globosa* LIP1), is a mono‐ and diacylglycerol lipase with an atypical loop‐like lid domain. Activation of SMG1 was proposed to be solely through a gating mechanism involving two residues (F278 and N102). However, through disulfide bond cross‐linking of the lid, this study shows that full activation also requires mobility of the lid domain, contrary to a previous proposal. The newly introduced disulfide bond makes lipase SMG1 eligible as a ratiometric thiol/disulfide redox potential probe, when it is coupled with chromogenic substrates. This redox‐switch lipase could also be of potential use in cascade biocatalysis.

**Abbreviations**
DAG: diacylglycerol
DTT: dithiothreitol
GSSG: glutathione oxidized
MAG: monoacylglycerol
pNPC: *p*‐nitrophenyl octanoate
TAG: triacylglycerol
TCEP: tris(2‐carboxyethyl)phosphine
WT: wild‐type

Lipases are a large group of enzymes capable of promiscuous reactions, including the well known hydrolysis and esterification of lipids, as well as the less known reactions such as C–C bond formation, C–heteroatom bond formation, and oxidative processes 1.

Lipase SMG1 from *Malassezia globose* CBS 7966 2, or *Malassezia globosa* LIP1, is a mono‐ and diacylglycerol lipase that does not accept triacylglycerides (TAGs), but only mono‐ and diacylglycerides (MAGs and DAGs) as substrates, which seems to be related to the specific hydrophobic interactions near the catalytic site 3. In addition to being an antidandruff target 4, lipase SMG1 is also a versatile biocatalyst and has been extensively characterized in terms of DAG production 5, phospholipids modification 6, as well as epoxidation of alkenes 7.

Crystal structures of SMG1 have been solved both in the apo‐enzyme form (closed, PDB: 3UUE) 8 and in complex with product (open, PDB: 4ZRD) 9. Aside from the canonical α/β hydrolase fold, catalytic triad and oxyanion hole, SMG1 has a unique lid fragment (T101‐D119) in the form of a loop, while most lipases have α‐helical lids. In most cases, lid domain is responsible for regulating the catalytic activity of lipase through conformational changes, it can either shield the catalytic site from the substrate by hovering over the catalytic pocket, or it can activate the lipase by moving away. Many studies have been devoted to the elucidation of lipase activation process at a molecular level 10, 11, gaining insight into the plasticity of the key structural regions relevant to activation, in an effort to offer guidelines for developing highly performing biocatalysts better suited to industrial needs. Previous studies identified that residue 102 within the lid of lipase SMG1 and residue 278 function as a gate, which regulates the active and inactive states of the lipase 9. Since no structural discrepancy was observed for the lid domain between the product‐bound open structure and the closed form, plus the relatively low B‐factor, the rest of the lid in lipase SMG1 was ascribed to be stable during activation.

To further validate whether this is indeed the case, we cross‐linked the lid to the main scaffold of lipase SMG1 by introducing a disulfide bond that could be reversibly controlled through reduction or oxidization. This design allows us to investigate whether the mobility of the lid is absolutely necessary for the activation of lipase SMG1.

## Materials and methods

### Chemicals

Endoproteinase trypsin (modified, sequencing grade) was obtained from Promega (Madison, WI, USA). TCEP and GSSG were obtained from Aladdin (Shanghai, China). DTT was purchased from Sangon (Shanghai, China). All other chemicals were purchased from Sigma (St. Louis, MO, USA).

### Protein expression and purification

The proteins were expressed in *Pichia pastoris*. SMG1‐WT was expressed with no tag and purified with anion‐exchange chromatography 12, while the mutant was expressed as a C‐terminal His‐tagged enzyme and purified by immobilized metal chromatography 3. The C‐terminal his tag has been shown to have no influence on the activity of the enzyme 13.

### Disulfide bond mapping using NanoLC‐ESI‐MS/MS

The lipase was denatured with 6 m Guanidine hydrochloride, and alkylated with iodoacetamide (50 mm, pH 8.0) to avoid possible formation of disulfide bonds between free cysteines. The sample was then desalted by dialysis, followed by the proteolytic digestions with trypsin in 100 mm NH_4_HCO_3_, pH 8.0 at 37 °C for 2 h. The digested sample was then split into two fractions of equal volumes. Fraction one was analyzed by NanoLC‐ESI‐MS/MS directly. Fraction two was reacted with 5 mm of DTT first, followed by reaction with 5 mm of iodoacetamide before being subjected to NanoLC‐ESI‐MS/MS. The data from two fractions were analyzed with ProtTech s2map software (ProtTech Inc., Phoenixville, PA, USA) package to identify the amino acid sequences of disulfide bond‐linked peptides. The identified hits were then validated manually.

NanoLC‐ESI‐MS/MS analysis of digested protein samples was carried out on HPLC system (Agilent, Santa Clara, CA, USA) using a reverse phase C18 column (80 mm × 7.5 mm i.d., 3‐μm particle size). Solvent A was composed of 97.5% water, 2% acetonitrile, 0.5% formic acid. Solvent B was of 9.5% water, 90% acetonitrile, 0.5% formic acid. The linear gradient from 2% Solvent B to 90% solvent B in 60 min was used at a flow rate of 800 nL·min^−1^.

The HPLC system was coupled on‐line with a Quadrupole ion trap mass spectrometer (Thermo, Palo Alto, CA, USA) in a way that samples eluted from the HPLC column were directly ionized by an electrospray ionization (ESI) process and entered into the mass spectrometer. The ionization voltage was optimized (within 1.2–1.8 kv). The capillary temperature was set at 110 °C. The mass spectrometer was set at the data‐dependent mode to acquire MS/MS data via a low‐energy collision‐induced dissociation (CID) process. The default collision energy was 33% and the default charge state was 3. One full scan with 1 microscan with a mass range of 550–1800 amu was acquired, followed by one MS/MS scan of the most intense ion with a full mass range and three microscans. The dynamic exclusion feature was set as following: repeat count of 1 within 0.3 min and exclusion duration of 0.4 min. The exclusion width was 4 Da.

### Lipase assay using pNPC

Oxidization of the mutant was done through full DTT reduction (10 mm) for 1 h, followed by oxidization with 50 mm of GSSG for 3 h, while buffered at the same time with 100 mm sodium phosphate, 0.1% gum arabic, pH 6.0. Reduction treatment of lipases was done by incubation with 10 mm of TCEP for 10 min, while buffered with 100 mm PIPES, 0.1% gum arabic, pH 6.7 instead, since TCEP is not compatible with phosphate buffer 14. The reducing effect of TCEP and DTT are equivalent in this case, DTT (longer treatment required) was used in GSSG‐oxidizing treatment for its reversibility. The reaction mixture contained 2 μL of treated/untreated lipase, 88 μL buffer (phosphate buffer for untreated/oxidized lipase, PIPES buffer for reduced lipase), and 10 μL of 1 mm pNPC in ethanol. The end‐point assay performed at room temperature was terminated by 0.1 m Tris, 1% SDS after 3 min. Relative activities were derived from specific activities using the activity of untreated WT as 100%.

### Lipase assay using DAG

Diacylglycerol of 1 g was emulsified in 9 mL of the phosphate buffer described above supplemented with 1% polyvinyl alcohol. Lipase SMG1 (2 mg·mL^−1^) was reduced with 10 mm of DTT on ice for 3 h; oxidization was done through further incubation with 50 mm of GSSG. DAG hydrolysis was carried out as described before 9. The reaction system contained 2.5 mL of DAG emulsion (about 0.25 g DAG) and 0.1 mg lipase in 0.5 mL of 100 mm phosphate, 0.1% gum arabic, pH 6.0, and was conducted at 30 °C. Relative activities were derived from specific activities using the activity of untreated WT as 100%.

## Results and Discussion

### Disulfide bond mapping of mutant L106C/V233C

**Figure 1:**
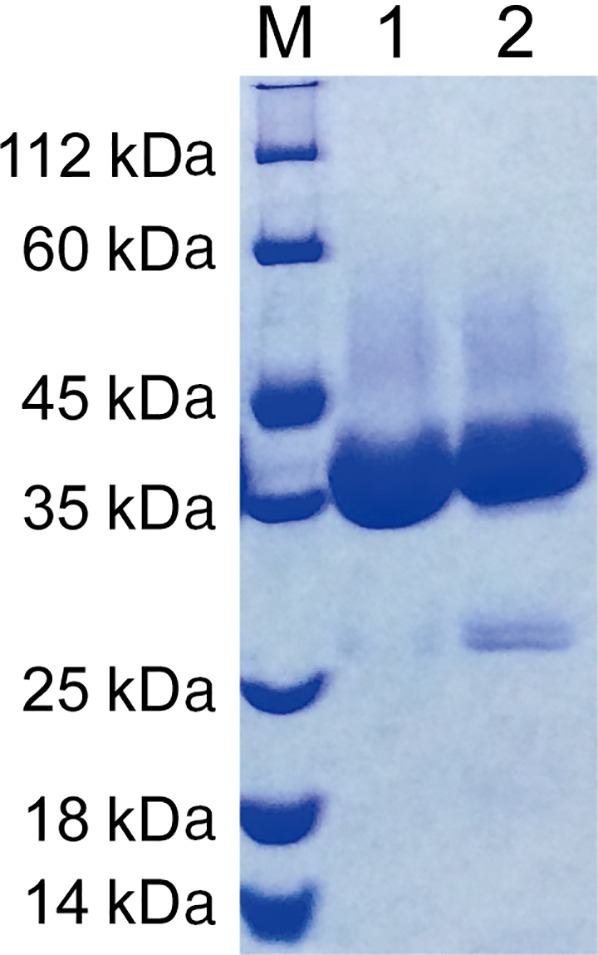
SDS/PAGE of SMG1‐L106C/V233C. Lane 1, L106C/V233C without DTT treatment; Lane 2, L106C/V233C treated with 10 mm DTT.

**Figure 2:**
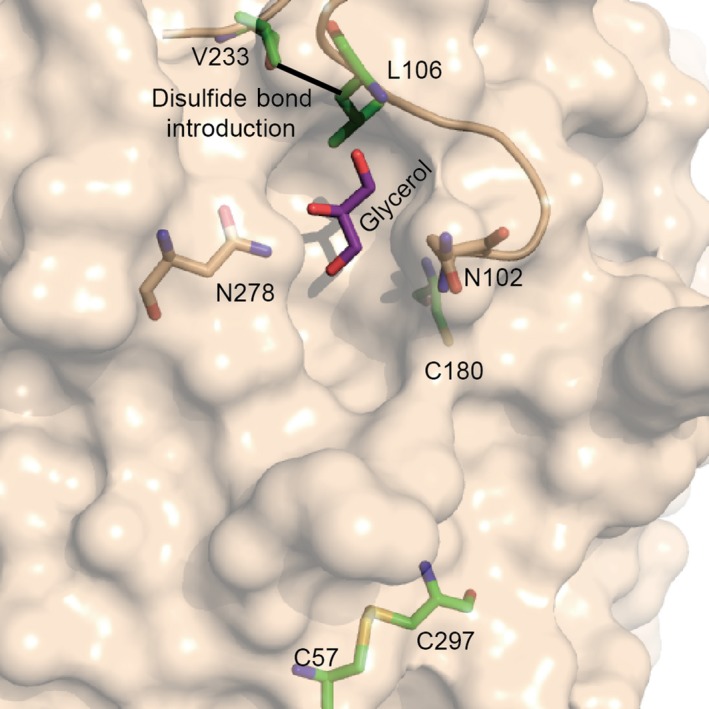
Glycerol‐bound structure of SMG1‐F278N (PDB: 4ZRD) with relevant residues and glycerol shown in sticks. Disulfide bond to be introduced between residues 233 and 106 is labeled with a black line.

**Figure 3:**
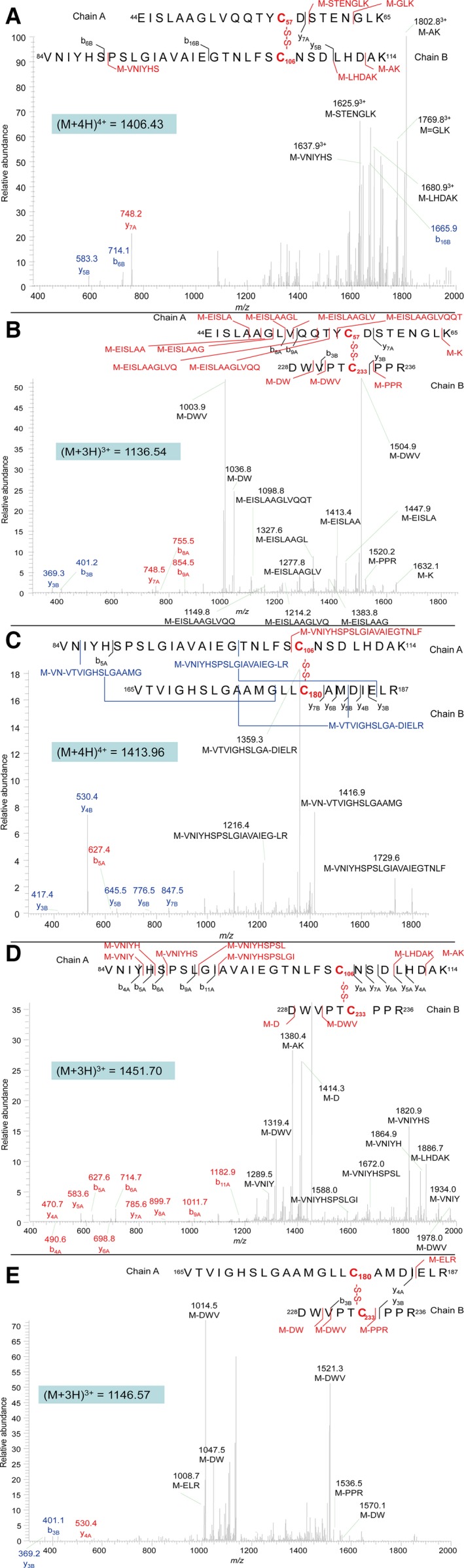
MSMS spectra of the disulfide linked peptides P4–P7 (A), P4–P24 (B), P7–P18 (C), P7–P24 (D), and P18–P24 (E), corresponding to disulfide bonds between C57–C106, C57–C233, C106–C180, C106–C233, and C180–C233, respectively. Disulfide bonds linking Chain A and Chain B are indicated in red. The sequence‐specific ions are labeled as ynA, bnA for Chain A, and ynB, bnB for Chain B on the spectrum. M indicates Chain A plus Chain B.

Upon close inspection of the crystal structure of SMG1, positions 106 and 233 were identified to be close enough for disulfide bond introduction, hence a double mutant L106C/V233C was constructed and purified. No dimer was detected by SDS/PAGE under oxidized/reduced conditions (Fig. 1). However, since a native disulfide bond (C57–C297) and a free cysteine exist in the wild‐type lipase SMG1 (Fig. 2), NanoLC‐ESI‐MS/MS was used to map the disulfide bonds within mutant L106C/V233C. Unfortunately, as shown in Table 1, only 31.5% of the disulfide bonds were formed as intended between C106 and C233, leaving roughly two‐thirds of the protein with mismatched disulfide bonds and thus misfolded and inactivated (mass values for trypsin digested fragments under reduced condition are shown in Table 2, while the MSMS spectra of the disulfide linked peptides are shown in Fig. 3.). Nevertheless, given the soluble nature of mutant L106C/V233C, refolding could be used to regain the natural conformation and the right match of disulfide bonds, with the help of a pair of redox‐shuffling agents, such as DTT/GSSG 15.

**Table 1:**
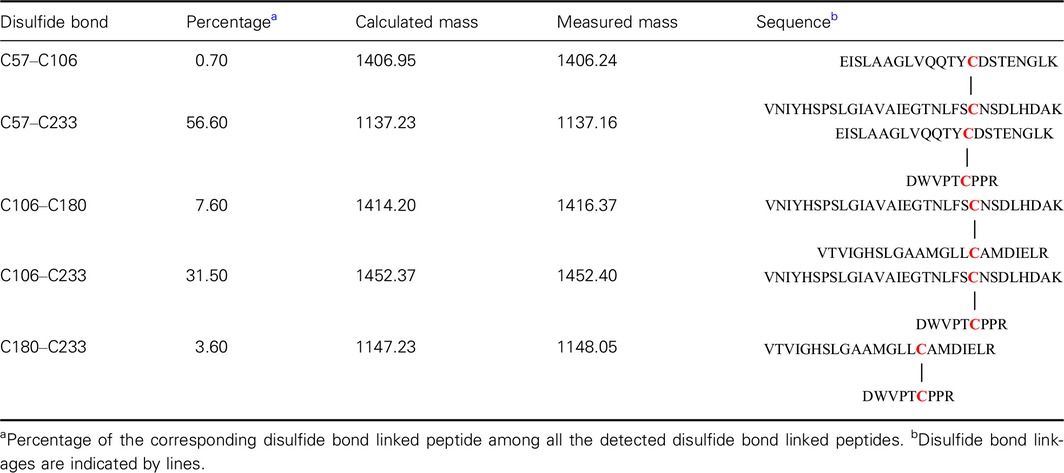
Disulfide mapping of SMG1‐L106C/V233C

**Table 2:**
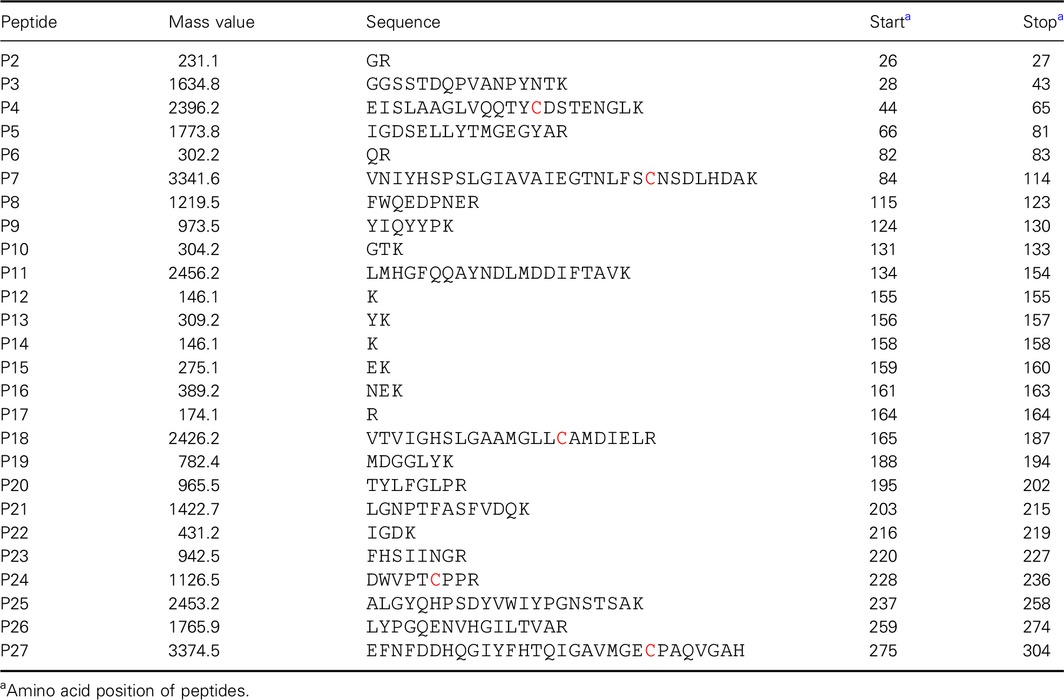
Calculated mass values for tryptic digests of SMG1‐L106C/V233C, reduced with DTT

### Lid mobility is required for full activation of lipase SMG1

**Figure 4:**
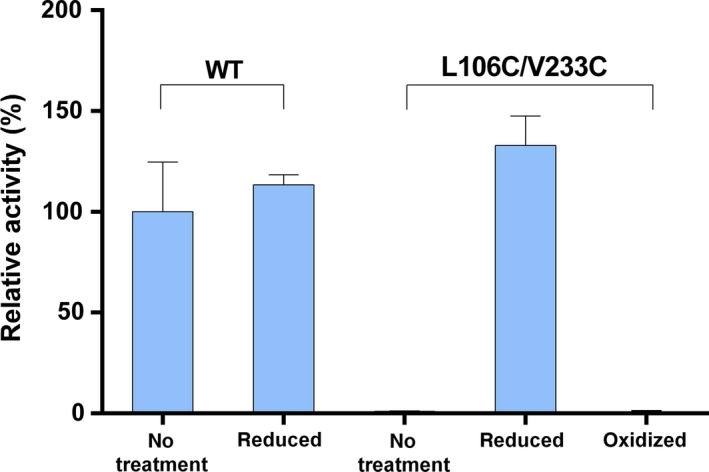
Relative activities of lipase SMG1 and mutant in oxidized and reduced form, using pNPC as substrate. All lipases were reduced with TCEP, oxidization of mutant L106C/V233C was done through reduction with DTT first, followed by oxidation with GSSG. Error bars stand for standard deviation. No significant differences were found between reduced WT or reduced L106C/V233C and untreated WT.

To investigate whether the mobility of the lid is absolutely necessary for activation, lipase activities of the mutant against pNPC were measured under reduced and oxidized conditions, which correspond to the free and cross‐linked states of the lid domain, respectively. DTT (slow, reversible) or TCEP (fast, irreversible) was used for reduction 14, while oxidization was achieved through reducing/refolding followed by GSSG treatment. As shown in Fig. 4, neither activity of the reduced WT or L106C/V233C was significantly different from that of the untreated WT, while the oxidized mutant was completely inactive.

More specifically, three conclusions could be drawn from these results. (a) Breaking the native disulfide bond between C57 and C297 does not affect the specific activity significantly, as seen from the data of untreated and reduced WT. (b) The specific activity of reduced L106C/V233C (no disulfide bond in between) is almost equivalent to that of the WT, indicating that the misfolded protein can recover to the natural conformation, which lays the foundation for protein refolding and disulfide bond rematching. This also shows that mutagenesis of these two residues into cysteine does not affect SMG1's activity against pNPC. (c) Full recovery of L106C/V233C to natural conformation after reducing implies that further oxidization treatment with GSSG would facilitate the rightly matched disulfide bonds between 106 and 233. This newly oxidized mutant is also inactive, like the untreated/oxidized L106C/V233C with specific activity two orders of magnitude below that of the wild‐type, concluding that when the rest of the lid domain is anchored, the lipase becomes inactive.

We have shown that mutation of residues 106 and 233 into cysteine does not change the activity of lipase SMG1 *per se*, but cross‐linking the cysteines together inactivates the lipase completely. That is, when the lid fragment is anchored to the main scaffold, the lipase in almost inactive; while when the lid is freed, the lipase is active again, as with the wild‐type. This indicates that the mobility of the lid domain is required for lipase SMG1 to function, and that in addition to the gating motion of residues 278 and 102, full activation of lipase SMG1 also requires the conformational change in the lid, which is probably related to the adsorption of SMG1 onto the hydrophobic phase of substrates. It is also possible that lid movement is necessary for the correct formation of oxyanion hole that is misformed in the closed‐lid crystal structures 16, 17.

**Figure 5:**
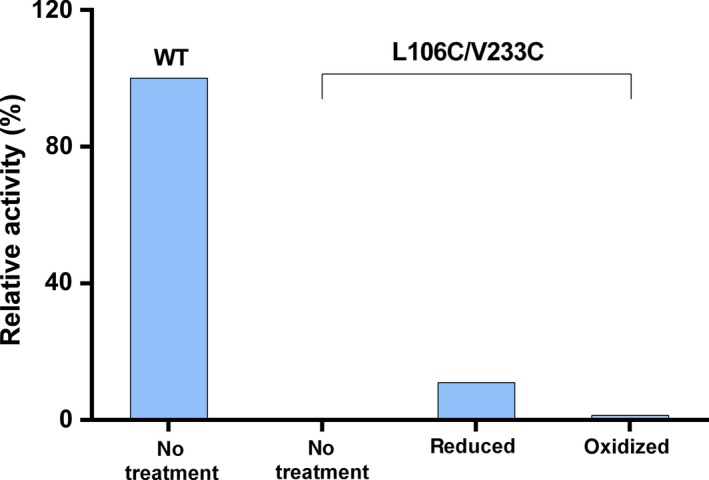
Relative activities of lipase SMG1 and mutant in oxidized and reduced form, using DAG as substrate. Experiments were performed in duplicate and deviation was within 5%.

Lipase assay using DAG as substrate showed similar results (Fig. 5), specific activity of the oxidized mutant was two orders of magnitude below that of the WT (about 1% relative to WT, possibly from none complete oxidation); however, activity of the reduced mutant restored to only about 10% of the WT activity. This is in contrast to the complete recovery of pNPC activity. Clearly, mutations of 106 and 233 changed the specificity of SMG1. One possible explanation is that mutation into less lipophilic residues is unfavorable for the binding onto the DAG/water interface. Residue 103 and 104 on the lid domain were demonstrated to help anchor the lid to the lipophilic phase of DAG 13, thus it is reasonable to suggest that mutation of 106 and 233 into less lipophilic residues would also impair binding of the lid region onto DAG phase. While this could affect the DAG activity, it will not affect pNPC activity because under the working concentration (0.1 mm) pNPC is soluble in aqueous phase with no defined interface.

These results reveal the pitfalls of investigating enzyme activation through crystallization. As useful as the active form crystal structure can be, it only presents static configuration and thus is unable to reveal the possible conformational change leading to the crystalized form. This could be misleading especially when the active form regains or recovers part of the initial inactive conformation.

### Potential application of mutant L106C/V233

A disulfide bond has been engineered into green fluorescent proteins (GFPs) or yellow fluorescent proteins (YFPs) to develop redox potential probes, that, upon equilibrium with the ambient thiol/disulfide compounds, could be used to measure redox potentials in cells in real time 18, 19. The L106C/V233 mutant described here is responsive to ambient thiol/disulfide redox potential change. Given the distinct activity difference between the reduced and oxidized form, the mutant could serve as a thiol/disulfide redox potential probe when coupled with chromogenic substrates such as pNPC, using initial velocity as signal output. Of course probes derived from this mutant would have to be used *in vitro*, but compared to the fluorescent signal measurement, only basic instrumentation is required; and unlike rxYFPs 18, the probe would be ratiometric in that the initial velocity signal reflects directly [SMG1]_red_/[SMG1]_ox_.

Furthermore, the redox potential readout range of the probe could be modified through tuning its thermodynamic mid‐point potential, which can be achieved through protein engineering, either by altering the pKa value of the disulfide bond in the probe 20, or by modifying the internal strain on the bond 21. Computational calculation of electric potential could also offer additional information to guide the rational engineering process 22.

The mutant described here is a redox‐switch lipase, containing a specific kind of switch for its lipase activity that could be of potential use in cascade biocatalysis, where different lipase activities were expected in multiple reaction steps involving different redox potentials.

## Conclusion

Through disulfide cross‐linking of the lid, it was proved that in addition to the gating motion of residues 278 and 102, full activation of lipase SMG1 also requires the conformational change in the lid domain. The newly introduced disulfide bond makes lipase SMG1 eligible as a ratiometric thiol/disulfide redox potential probe when coupled with chromogenic substrates, as well as to be of potential use in cascade biocatalysis.

## Author contributions

SG designed and carried out the experiments, and wrote the manuscript. GMP conceived the initial project and revised the manuscript. DL performed the DAG hydrolysis assay. DY revised the manuscript. YW supervised the project and approved this manuscript.
